# Curvature Constrained Splines for DFTB Repulsive Potential Parametrization

**DOI:** 10.1021/acs.jctc.0c01156

**Published:** 2021-02-19

**Authors:** Akshay Krishna Ammothum Kandy, Eddie Wadbro, Bálint Aradi, Peter Broqvist, Jolla Kullgren

**Affiliations:** † Department of Chemistry - Ångström Laboratory, Uppsala University, Box 538, 751 21 Uppsala, Sweden; ‡ Department of Computing Science, Umeå University, Umeå SE-901 87, Sweden; ¶ Bremen Center for Computational Materials Science, University of Bremen, Am Fallturm 1, 28359 Bremen, Germany

## Abstract

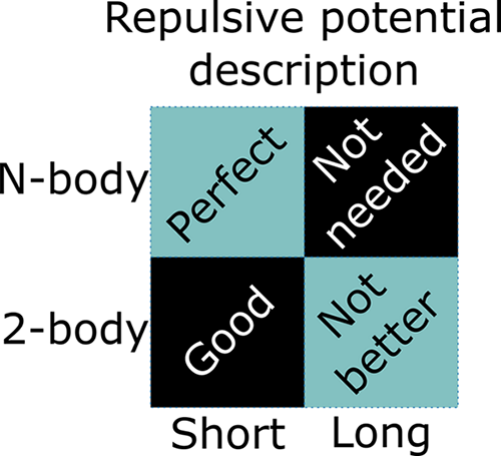

The Curvature Constrained Splines (CCS) methodology has been used for fitting repulsive potentials to be used in SCC-DFTB calculations. The benefit of using CCS is that the actual fitting of the repulsive potential is performed through quadratic programming on a convex objective function. This guarantees a unique (for strictly convex) and optimum two-body repulsive potential in a single shot, thereby making the parametrization process robust, and with minimal human effort. Furthermore, the constraints in CCS give the user control to tune the shape of the repulsive potential based on prior knowledge about the system in question. Herein, we developed the method further with new constraints and the capability to handle sparse data. We used the method to generate accurate repulsive potentials for bulk Si polymorphs and demonstrate that for a given Slater-Koster table, which reproduces the experimental band structure for bulk Si in its ground state, we are unable to find one single two-body repulsive potential that can accurately describe the various bulk polymorphs of silicon in our training set. We further demonstrate that to increase transferability, the repulsive potential needs to be adjusted to account for changes in the chemical environment, here expressed in the form of a coordination number. By training a near-sighted Atomistic Neural Network potential, which includes many-body effects but still essentially within the first-neighbor shell, we can obtain full transferability for SCC-DFTB in terms of describing the energetics of different Si polymorphs.

## 1 Introduction

Within the field of material science, Density Functional Theory (DFT)^1,2^ has become one of the main working horses owing to its wide range of applicability and its favorable scaling behavior with system size. Despite its success, DFT is not computationally efficient for systems containing a large number of atoms, sampling of complex energy landscapes, and for high-throughput screening purposes.^3^

In regard to challenges with computational efficiency, a force-field (FF) based approach would be ideal. However, the lack of an electronic description makes these approaches unable to estimate electronic properties (e.g., band structure, band gap, etc.). In general, the gap between DFT and FF-methods is filled by semiempirical methods, which strikes the right balance between accuracy and computational cost.

Self-consistent charge density functional tight binding (SCC-DFTB)^4^ is a semiempirical method, an approximate and parametrized DFT method, about 2 orders of magnitude faster than DFT when using local or semilocal density functionals. Compared to hybrid density functionals, the gain is even larger. The method is applicable to a wide range of problems within chemistry and physics, including redox chemistry of oxides,^6^ van der Waals interactions,^7^ electron transport,^8^ etc. The method can also be systematically extended to overcome limitations in DFT, such as the problem with underestimated band gaps.^9,10^

While SCC-DFTB is a very capable tool that can be used to calculate both geometries and electronic properties at a relatively low computational cost, there is a rather substantial effort required in terms of parametrization when applied to new systems. There are primarily two types of parameters in the SCC-DFTB method: (i) those related to the electronic structure and electronic energy of the valence electrons, the so-called compression radii, and (ii) those related to the repulsion between the ionic cores (the core electrons and the nuclei). Generally, the electronic parameters are optimized first to obtain an accurate electronic structure description. Thereafter, empirical short ranged two-body potentials are used to correct for the remaining missing contributions. We will refer to these potentials as repulsive potentials. Ideally, these potentials should be monotonic, smooth, and not vary too rapidly.^11^ Moreover, transferability is limited, due to the pairwise additive nature of the repulsive potentials, which means that when we extend beyond the range for which a particular parameter set was developed, we must be prepared to reparametrize the potential. Hence, the parametrization of the repulsive potentials is often a laborious and tedious task, calling for the development of novel methods utilizing robust, efficient, and fast mathematical routines to make the generation of reliable parameter sets more efficient.

In recent years, a number of initiatives to develop automated fitting procedures and protocols have been developed (see, e.g., refs (9 and 11−26)). Some of these focused exclusively on obtaining the parameters for the repulsive potential.^12−16,18,21,23,25,26^ A key feature among these developments is the use of splines to represent the repulsive potential. Here, the initial steps toward a semiautomated repulsive potential fitting scheme was done by Knaup et al.^12^ by their use of cubic splines combined with an evolutionary algorithm to find the best repulsive potential. A more rigorous least-squares optimization using fourth-order splines was done by Gaus et al.^14^ A drawback with this procedure was the difficulty in determining the knot position of the splines. Later, Chou et al.^9^ presented an automated Multi-Objective Particle Swarm Optimization (MOPSO) approach for optimizing both the knot positions of the splines for the repulsive potential as well as electronic parameters in the form of *l*-dependent compression radii. However, even though splines satisfy the criteria of smoothness, their flexibility can in some cases lead to oscillations in the repulsive potential. This prompted Bodrog et al.^13^ to instead define the repulsive potential as a linear combination of higher-order polynomials (excluding zeroth and first-degree terms) or of exponential basis functions . Indeed, the exponential basis functions (monotonous and smooth) are an ideal choice to approximate the repulsive potential, but in reality, the repulsive potential need not always be strictly repulsive. Therefore, the rigid exponential form for the repulsive potential becomes a drawback. Furthermore, the user has to identify the choice of basis functions required for a specific system, which could be problematic in some cases.

On a similar note, Hellström et al.^16^ found that the problem of incorrect energetics for various polymorphs of ZnO using the znorg-0.1 parameter set by Moreira et al.^27^ could be alleviated by a reparametrization of the repulsive potential using a training set of structures with different coordination numbers. For the new repulsive potential, a four-range Buckingham potential was chosen, an analytical function which the authors found to constitute a fair balance between flexibility and smoothness. The advantage of such an approach is that it avoid problems with oscillations that can occur for splines or high order polynomials. Clearly, the rigid parametric functional form of the four-range Buckingham is a drawback. More recently, Panosetti et al.^26^ introduced a Gaussian Process Regression (GPR) machine learning approach for fitting the two-body repulsive potential. Even though GPR is a nonparametric approach, the optimization of hyperparameters can be a challenge. Another problem is that GPR models require a large number of data points to train the potential against and are known to have poor extrapolation capabilities.

In this work, we demonstrate how the curvature constrained cubic splines (CCS) methodology developed in ref (28) can be used to build repulsive potentials without the need for adjustable parameters. The aforementioned problems with splines (oscillatory behavior and nonmonotonocity) are alleviated by defining a set of intuitive constraints on the curvature of the repulsive potential. An additional benefit of using the CCS methodology is that the optimization problem, i.e., the actual fitting of the repulsive potential, is a convex problem which can be solved using quadratic programming, which guarantees a unique (if strictly convex) and optimum two-body repulsive potential in a single shot.

The methodology is tested for various polymorphs of Si. We examine the flexibility of the CCS method in terms of adopting different shapes to best reproduce the energetics for a number of silicon polymorphs (3D and 2D). Since the CCS method is virtually free from meta-parameters, apart from the cutoff radius in the two-body interaction, it reduces the number of parameters in a SCC-DFTB parametrization to merely the electronic ones. As such, the CCS method is ideal to use in conjunction with global optimization techniques like the MOPSO method presented in ref (9).

The outline of the paper is as follows: In Section 2, we first briefly introduce the SCC-DFTB formalism and the CCS methodology and show in detail how it can be used for fitting the repulsive potential. Then, in Section 3, we describe the computational details concerning our DFT and SCC-DFTB calculations. In Section 4, we discuss the results obtained, and Section 5 concludes the paper.

## 2 Theory

### 2.1 DFTB

The SCC-DFTB method is based on a Taylor expansion of the Kohn–Sham energy functional about a reference density, ρ_0_, taken to be a superposition of pseudoatomic densities.^4^ The total energy expression in SCC-DFTB is normally truncated at the second-order and thus becomes
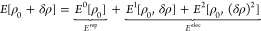
1*E*^1^ is obtained from the occupied eigenstates. More precisely, it is computed by
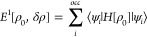
2By the use of a linear combination of atomic orbitals (LCAO) ansatz and a two-center approximation, all required entries for solving eq 2 can be conveniently precalculated and stored in so-called Slater–Koster tables. The second-order term, *E*^2^, describes the energy originating from density fluctuations about the reference ρ_0_. The term *E*^0^ primarily describes the ionic core–ionic core repulsion. However, the term essentially includes all remaining energy contributions not captured by the other two terms. The total repulsive energy of a system in SCC-DFTB is a sum of contributions of repulsive potentials *V*_rep_(*r*) from each atom pair
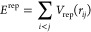
3where *i* and *j* run over the atom indices in the system, and *r*_*ij*_ is the distance between pair of atoms. The *V*_rep_ is usually short-ranged and smoothly decaying to zero at a certain cutoff distance (*r*_cut_). For a comprehensive description of the SCC-DFTB method and its capabilities, we refer to refs (11) and^29^.

### 2.2 *V*_rep_ Using Curvature Constrained Splines

The repulsive potential in the actual SCC-DFTB is often constructed using cubic splines. Cubic splines are flexible and easy to use, and their coefficients can be optimized by performing least-squares fitting to reference values. Here, we use the Curvature Constrained cubic Splines (CCS) methodology to provide the best possible repulsive potential. The reason is that traditional cubic spline methods can lead to repulsive potentials with spurious oscillations due to overfitting unless a small number of knots (number of spline intervals) are used, but a small number of knots lead to a poor linear approximation of the repulsive potential’s Hessian. To improve the description of the Hessian, Gaus et al.^14^ suggested the fourth-order spline fitting (a quadratic approximation for the Hessian) as an alternative. However, the former approach, though an improvement, still needs manual intervention to decide the number of knots below which overfitting can be avoided. In the CCS methodology, constraints are imposed in such a way that overfitting is prevented irrespective of the number of knots used.^28^ Hence, the Hessian can be approximated with arbitrary accuracy by increasing the number of knots. Moreover, constraints can be applied to each spline coefficient to have various shape-preserving properties. Consider a pair of atoms, the repulsive potential (*V*_rep_) between these atoms is defined from an interval of interatomic distances *r* ranging from (0,*r*_cut_). We subdivide the interval into *N* subintervals *I*_*n*_ = [*x*_*n*–1_,*x*_*n*_], for *n* = 1, ..., *N*. On each subinterval, we define a cubic function
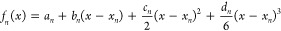
4To determine the so-called spline coefficients, we impose interpolation conditions for the second derivative of the spline and continuity conditions for the spline function itself as well as its first derivative. We remark that this treatment is different from the standard. A typical approach is to impose interpolation conditions on the spline function itself and the continuity conditions on its first and second derivatives. The reason for this treatment is that here we are interested in stipulating the curvature or the second derivative of the spline function at each subinterval’s end points. That is, we impose the 2*N* conditions
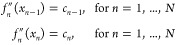
5Later, we will use the curvatures as the unknowns in an optimization problem to determine the best potential. (We remark that the above conditions ensure that *f*_*n*_^″^(*x*_*n*_) = *f*_*n*+1_^″^(*x*_*n*_) for *n* = 1, ..., *N* – 1.) Moreover, we impose the following continuity conditions
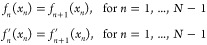
6at the interior interval end points. This gives us 2*N* – 2 additional conditions for the 4*N* spline coefficients. Finally, to close the system, we also require the spline to have zero value and gradient at the *x*_*N*_ = *r*_cut_, that is,
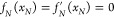
7By using the above relations (6 and 7), we can show that the coefficients ***a*** = [*a*_1_,*a*_2_,...,*a*_*N*_]^T^, ***b*** = [*b*_1_,*b*_2_,...,*b*_*N*_]^T^, and ***d*** = [*d*_1_,*d*_2_,...,*d*_*N*_]^T^ are linearly dependent on the imposed curvatures ***c*** = [*c*_0_,*c*_1_,...,*c*_*N*_]^T^.

We want to express the pair potential function *V*_rep_(*r*) as a spline function. So, in eq 3, the pair potential function *V*_rep_(*r*) is substituted with eq 4 and can be written as follows:
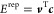
8A detailed derivation of vector ***v*** can be found in ref (28). In this work, we add an additional one-body term to the repulsive potential, to get the correct energies at the dissociation limits. This is shown below
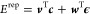
9where ***ϵ*** and ***w*** are the vectors containing one-body energy terms and number of atoms, respectively. The usage of one-body terms has earlier been found to improve geometries and reaction energies.^13^ In this work, the one-body terms were used to aid the fitting process and to investigate the lack of transferability of the two-body potential (see Section 4.5). The repulsive potential energy (*E*^rep^) has a linear dependence on the unknown coefficients ***c*** (see eq 8). As is detailed below, this implies that the coefficient vector ***c*** can be solved via the least-squares regression method. To get an accurate repulsive potential, the difference between the reference energies and corresponding DFTB electronic energies are minimized over a set of diverse chemical configurations ranging from *k* ∈{1,...,*K*}, where *K* denotes the number of configurations in the training set. The objective function (*J*) can be written down as follows
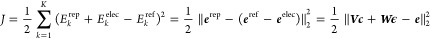
10where
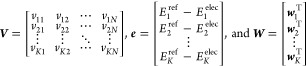
The ***W*** in eq 10 is a matrix for heteroatomic systems and a vector for homoatomic systems. On a similar note, ***ϵ*** is a vector for heteroatomic systems and a scalar for homoatomic systems. The objective function in eq 10 can be written as
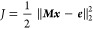
11where
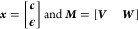
12The determination of the best vector ***x*** can be written as the standard Quadratic Programming (QP) problem
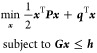
13where ***P*** = ***M***^T^***M*** and ***q*** = −***M***^T^***e***. The details for constraint matrices ***G*** and ***h*** are discussed in the next section. By construction, the matrix ***P*** in eq 13 is at least positive semidefinite, and hence the QP problem is convex. A convex optimization problem has the advantage that all local minima are global minima. If ***P*** is positive definite, then the problem is strictly convex, and thus only has one minimum.

### 2.3 Constraints

The highlight of the CCS method is that the shape of the optimized potential can be tuned via constraints on the curvature at the knot intervals. Additionally, the user is free to constrain the potential based on prior information about the system. We have developed new constraints exclusively for SCC-DFTB repulsive potential fitting (see Sections 2.3.1 and 2.3.3). For simplicity of notation, we omit constraints on ***ϵ***, so for the discussion below ***x*** = ***c***.

#### 2.3.1 Repulsive and Monotonous Constraints

**Figure 1:**
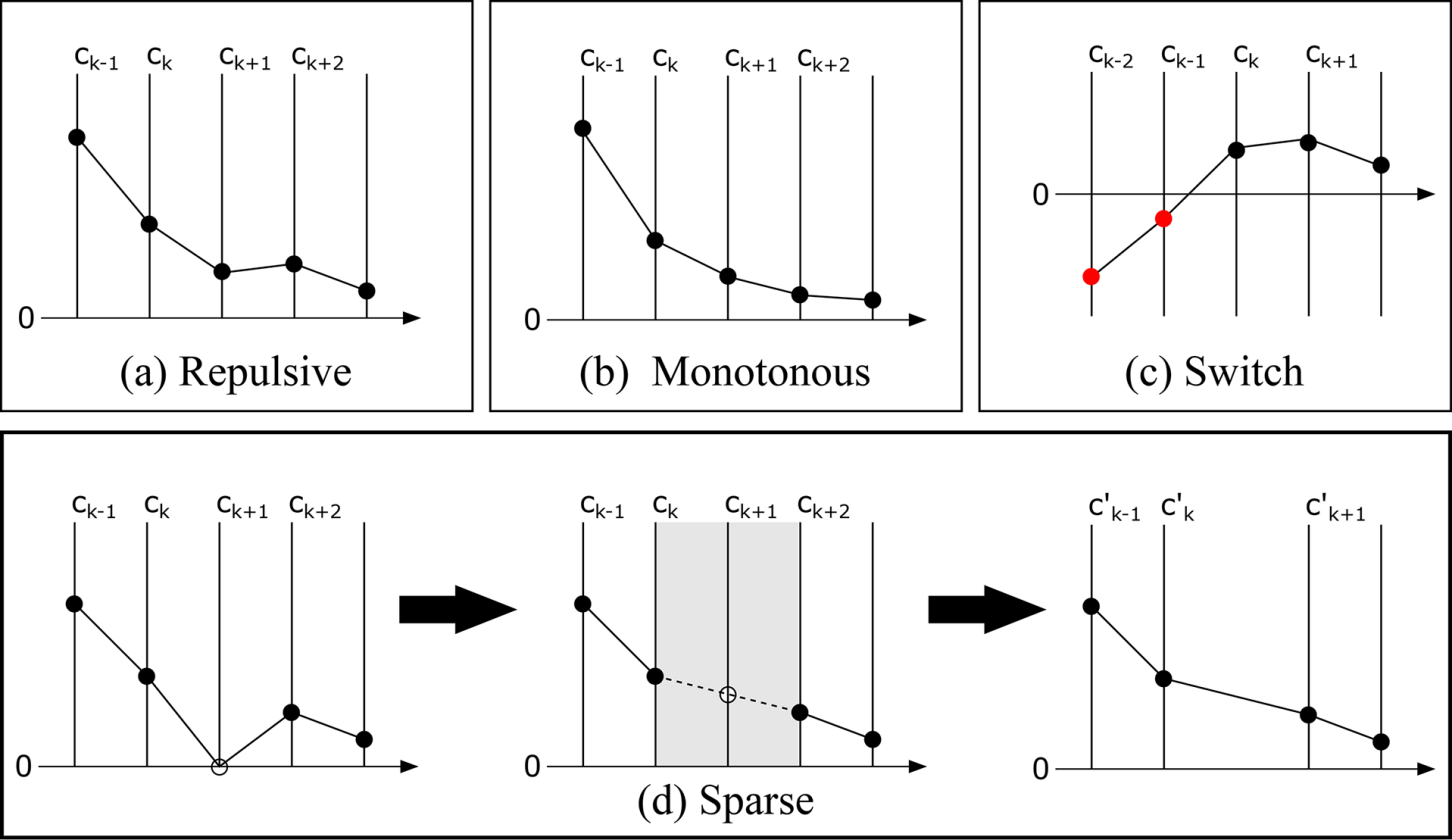
A schematic illustration of all the constraints used in CCS. The *x*-axis is divided into intervals, and on each interval we define a cubic spline uniquely determined by the ***c*** coefficients. The top panels (a) and (b) show the repulsive and monotonous constraints on ***c*** coefficients. Panel (c) depicts the switch constraint with red and black dots, respectively, indicating negative and positive values for ***c*** coefficients. The switching point *N*_switch_ is at the *k*th knot. The bottom panel (d) represents the sparse constraint. The knot point with an open circle indicates a bin with no data points. The ***c*** coefficient of the spline here is undetermined and can without loss of generality be set to an average value of neighboring ***c*** coefficients. This is equivalent to merging of the bins or intervals.

The repulsive constraint ensures that the spline approximated repulsive potential has a strictly positive curvature. However, such repulsive potentials can still have oscillations in the second derivative which may lead to poor forces and frequencies. In such cases, it would be ideal to have a tighter set of constraints with monotonically decreasing curvature values (see Figure 1). The corresponding constraint matrices ***G*** and ***h*** (shown in eq 13) are given by
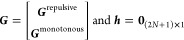
14in which **0**_(2*N*+1)×1_ is the zero matrix of dimension (2*N* + 1) × 1 and
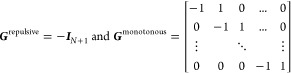
15where ***I***_*N*+1_ is the identity matrix of dimension (*N* + 1) × (*N* + 1), and ***G***^repulsive^ has dimension *N* × (*N* + 1).

#### 2.3.2 Switch Constraint

Due to the approximations in SCC-DFTB, the repulsive potential at times can have some attractive regions. This cannot be captured by the repulsive constraints discussed above. By instead adding a switch constraint, we allow the curvature values to change sign once (see Figure 1c) at a certain knot position called *N*_switch_. This allows the repulsive potential to have at most one minimum (for more details see ref (28)). The corresponding constraint matrices are given by
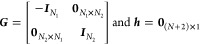
16where *N*_1_ = *N*_switch_, *N*_2_ = *N* + 1 – *N*_switch_, and **0**_*N*_1_×*N*_2__ is the zero matrix of dimension *N*_1_ × *N*_2_.

#### 2.3.3 Sparsity Constraint

The success of the CCS method lies in its flexibility, which can adopt the shape of the repulsive potential to arbitrary precision under the given constraints by gradually increasing the number of knots in the spline table. However, such a procedure can lead to a situation in which our problem becomes underdetermined, a problem of sparsity that needs to be handled. Thus, if we choose a fine mesh of knots, we can resolve the curvature in each part of the interval very well. However, in some regions where data is sparse, for example, at distances in between the first and second coordination shells, we have many more knots than there is information available. In other words, the curvature at certain knots cannot be uniquely determined. This problem can be handled by removing redundant knots by subinterval merging. The procedure is illustrated in Figure 1d and ensures that the curvature changes linearly over coherent undetermined subintervals. With this technique, we avoid any ambiguity in the optimization, and in the limit of an infinitely fine mesh, the method would correspond to one having freely adjustable knot positions.

## 3 Computational Details

Our primary reference method in the validation and testing of our new spline method is density functional theory in the implementation with plane waves and pseudopotentials. More specifically, the electronic wave functions were expanded in a plane-wave basis set with a kinetic energy cutoff of 600 eV. The core–valence interactions were modeled with pseudopotentials generated within the Projector Augmented Wave (PAW) scheme proposed by Blöchl.^30^ In the calculations, we explicitly treated four electrons for each Si atom. Furthermore, we used the PBE functional^31^ as a reference to generate a training set for repulsive potential fitting (i.e., energies). However, as semilocal DFT functionals generally give poor band gap estimates, we used a modified HSE06 functional^32,33^ (denoted as HSE06’) for this purpose. Instead of the normal 25% nonlocal Fock exchange, we used 10%, which previously has been shown to yield electronic band gaps in better accord to experiments.^34^ All DFT calculations were performed with the Vienna Ab-initio Simulation Package (VASP).^35−38^ All SCC-DFTB calculations were done using the DFTB+ software.^29,39^ The repulsive potential fitting was performed using a modified version of the CCS package.^28^

## 4 Results and Discussion

In this section, we will demonstrate some key features of CCS when used in conjunction with SCC-DFTB. We start by introducing our training set, which consists of various Si polymorphs. Before fitting the repulsive potentials, we start discussing transferability concerning electronic properties. Next, we demonstrate the flexibility of CCS in terms of adapting to different shapes when fitted to data for Si in different chemical environments, here expressed in terms of varying coordination numbers. We further utilize key features of the CCS method which allow us to address the question of the apparent lack of transferability of SCC-DFTB and propose possible solutions to this issue.

### 4.1 Structures and Training Set

**Figure 2:**
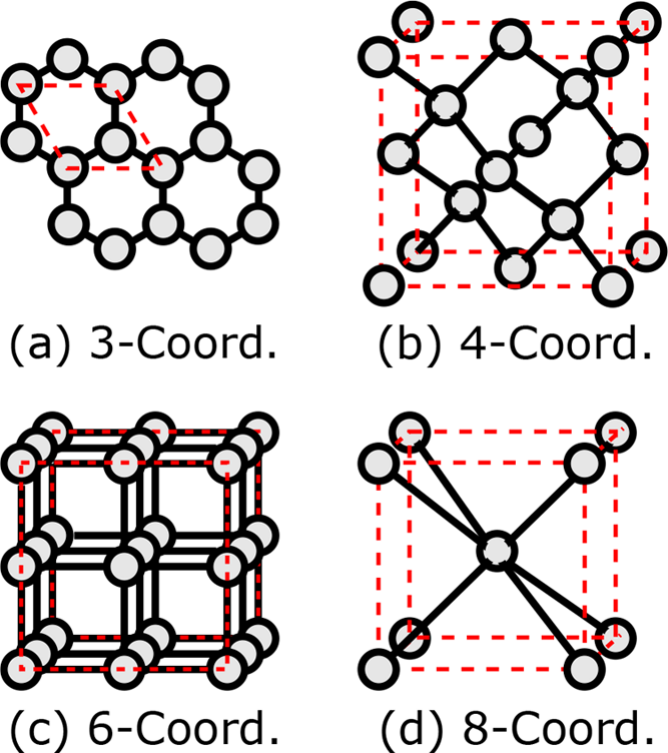
Diversity in the local chemical environment for Si polymorphs expressed in terms of a coordination number: (a) graphene, (b) diamond, (c) simple cubic, and (d) body-centered cubic.

As the training set, we have chosen crystalline phases of Si that lack internal parameters to ensure that we probe effects due to an isotropic chemical environment. There is, however, no technical hindrance to also add structures with internal parameters. The following polymorphs of Si were considered (with corresponding coordination numbers): graphene (3 coordinated), diamond (4 coordinated), simple cubic (6 coordinated), and body-centered cubic (8 coordinated). A schematic illustration of the polymorphs used is shown in Figure 2. The training set comprises Energy-Volume (E-V) scans for all the polymorphs. The volumes in the training set correspond to nearest-neighbor distances between Si from 2.1 to 3.3 Å in steps of 0.1 Å. The nearest-neighbor distance distributions for the different polymorphs are shown in Figure 4.

### 4.2 Transferability in Electronic Parameters

Before fitting repulsive potentials, the quality of the electronic parameters of existing Slater-Koster tables available in the SCC-DFTB community is validated by comparing computed electronic properties toward hybrid-DFT data. In the literature, the following Slater-Koster tables for Si are available: pbc-0.3,^40^ matsci,^41^ and siband.^10,42^ The pbc-0.3 and matsci sets are known to give a poor description of the band structure for Si polymorphs.^9^ In contrast, the electronic parameters of the siband set were optimized by Markov et al.^10^ toward experimental Si and SiO_2_ band structure data.

**Figure 3:**
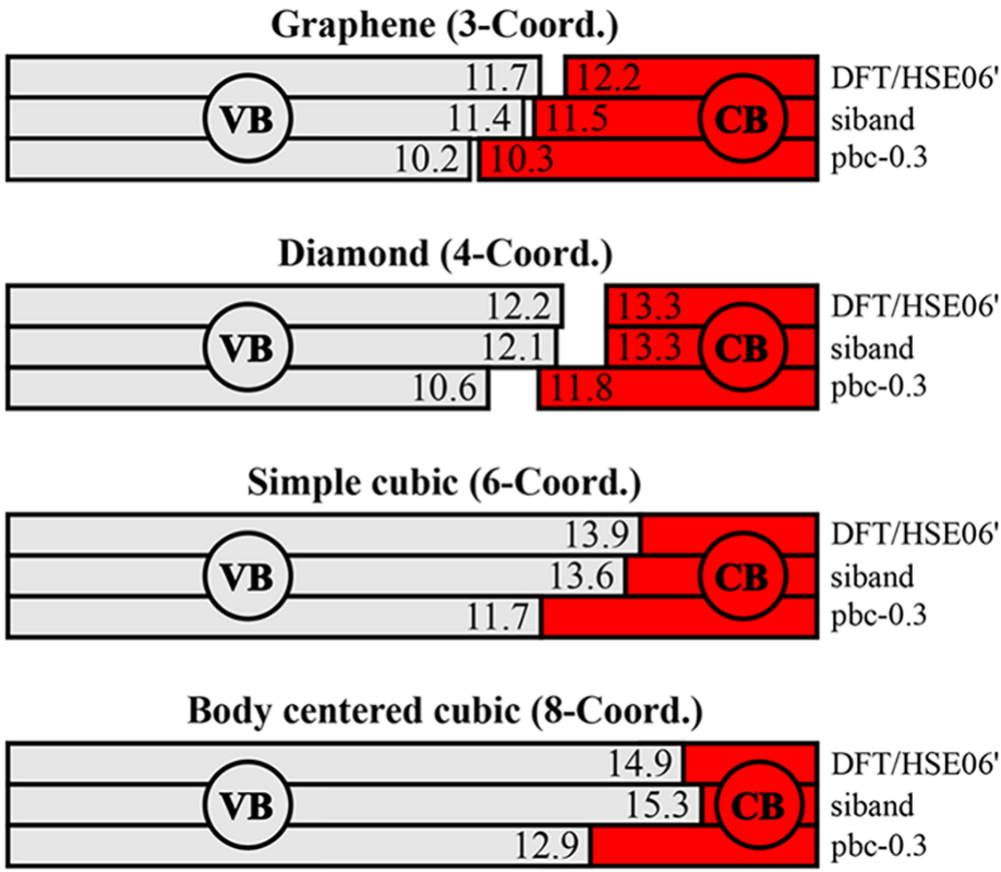
A schematic description of the electronic structure for Si polymorphs including graphene, diamond, simple cubic, and body-centered cubic. The valence band (VB) and the conduction band (CB) are colored gray and red, respectively. The gap between VB and CB for the nonmetallic polymorphs (diamond and graphene) indicates the band gap. All energies are in eV.

The electronic structures in terms of bandwidths and band gaps for the different Si polymorphs in our training set calculated using hybrid DFT and SCC-DFTB with pbc-0.3 and siband are shown in Figure 3. The energies in the plots are aligned through the lowest lying occupied Si state, i.e., the bottom of the valence band. We note that the bandwidths of the valence bands and conduction bands are in good agreement between our modified hybrid DFT calculations and the SCC-DFTB using the siband set for all polymorphs. From these data, it is further clear that the siband set is superior to the pbc-0.3 set and that the transferability when it comes to SCC-DFTB electronic properties, here in terms of bandwidth and band gap, is rather good.

### 4.3 Two-Body Hyperparameter *R*_cut_

**Figure 4:**
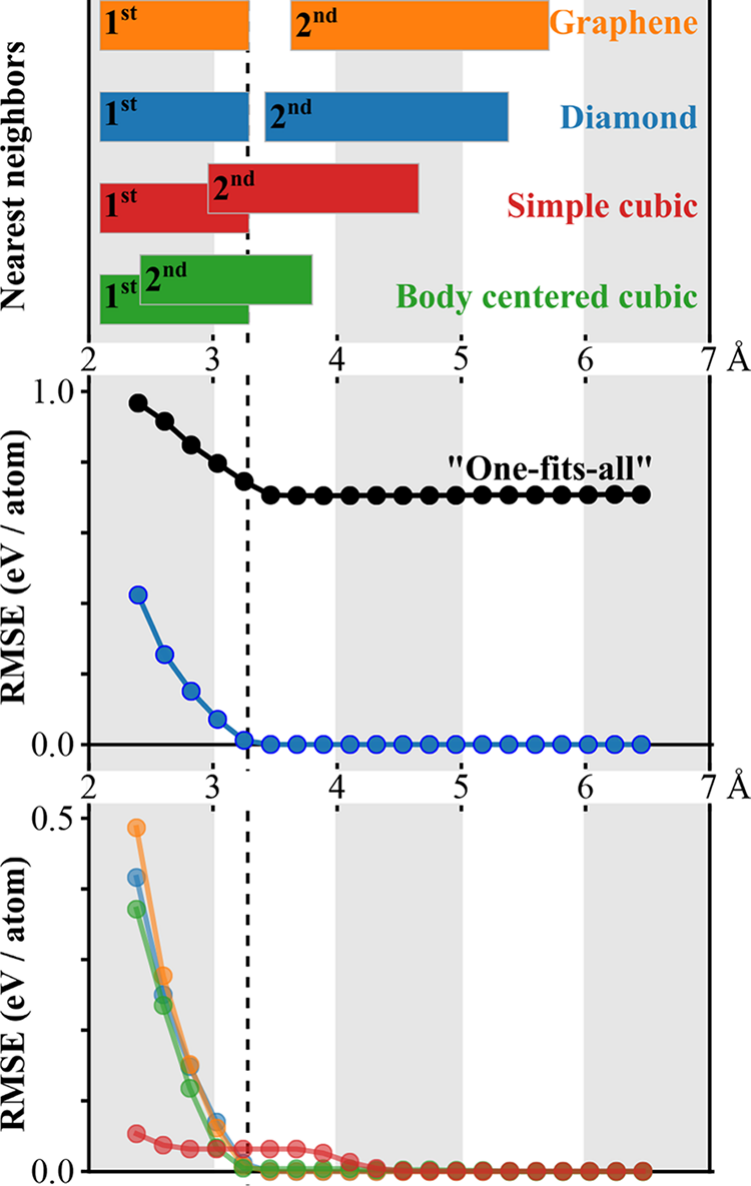
Top panel shows the range of first and second nearest-neighbor distances for graphene (orange), diamond (blue), SC (red), and BCC (green) in the training set. The middle panel shows the variation of RMSE as a function of *R*_cut_ for both diamond (blue) and all polymorphs (black). The bottom panel shows the variation of RMSE as a function of *R*_cut_ for individual polymorphs. The dashed vertical line at 3.3 Å indicates the largest nearest-neighbor distance in the training set.

Before generating the repulsive potentials using the CCS scheme, we need to determine the two-body hyperparameter *R*_cut_ and how the changes in this parameter affect the accuracy of the resulting potential. The repulsive potential in SCC-DFTB is usually short-ranged, and in general, we use a small cutoff value for *R*_cut_. Typically, the range of the first nearest-neighbor distances in the training set (refer to Figure 4 top panel) is used. However, using CCS the ideal cutoff could be determined from a simple grid search. For this purpose, we made the following training sets: *i)* a set containing E-V scans for all the polymorphs and *ii)* sets containing E-V scans for individual polymorphs. The *R*_cut_ values were varied from 2.38 to 6.42 Å, and optimization was performed using the switch constraint (see Section 2.3.2).

In Figure 4 (middle and bottom panels), we show the variation of the training set error as a function of the *R*_cut_ values. We infer that the training set error converges immediately after the first nearest-neighbor distances, except for the SC polymorph. The convergence occurs at 4.3 Å for the SC polymorph. Overall, this suggests that the assumption of a short-ranged behavior for the repulsive potential seems valid in this case. The root-mean-squared-error (RMSE) of individual polymorphs is less than 10^–2^ eV/atom at *R*_cut_ greater than 3.4 Å. Hence, we have chosen a *R*_cut_ value of 3.4 Å for the Si–Si repulsive potential. The training set error for individual polymorphs converges toward zero, whereas a nonzero convergence is seen for the training set including all polymorphs. A nonzero value for the training set error convergence indicates the limit of accuracy for the two-body approximation. We remark that the choice of electronic parameters might influence this value. In principle, for a given training set, one could search for a set of electronic parameters that minimize the converged error in the two-body approximation. This could be done by combining CCS with a global search algorithm, e.g., MOPSO.

### 4.4 Repulsive Potential Fitting Using CCS

**Figure 5:**
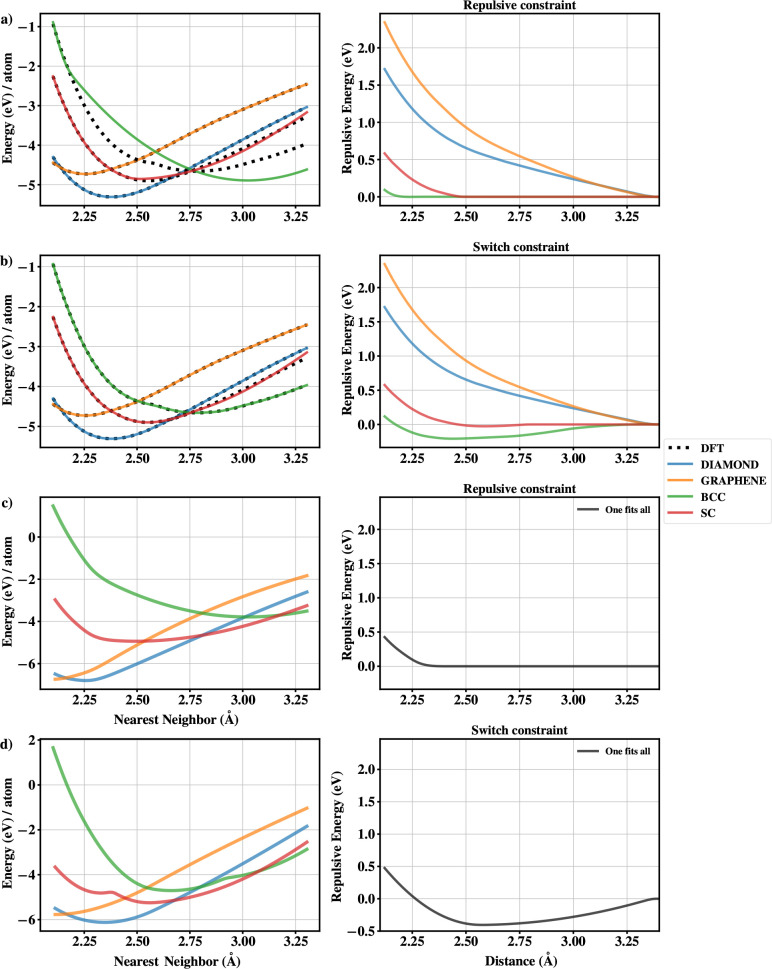
Left panel in a) compares DFT energies (black dotted lines) with SCC-DFTB for various polymorphs of Si, with a repulsive constraint. The corresponding repulsive potentials are shown to the right. Panels in b) show a corresponding comparison for the switch constraint. Panels c) and d) show the best approximate potential for all Si polymorphs with a repulsive and switch constraint, respectively.

Having established a scheme for obtaining the optimal *R*_cut_ value, we move on to discuss the generation of the repulsive potentials for the silicon polymorphs in our training set (see Section 4.1). The electronic energies from SCC-DFTB are obtained using the siband Slater-Koster tables of Markov et al.^10^ The CCS method was used to optimize the repulsive parameters. Two different types of fitting procedures (optimizations) were done, one using the original constraints on the curvature (strictly positive) and one in which the curvature is allowed to change sign once (switch constraint, see Section 2.3.2). The resulting repulsive potentials are shown in Figure 5.

Our first observation is that there is no good repulsive potential that can simultaneously reproduce the energetics for all the polymorphs in the training set with an acceptable accuracy. This is not completely unexpected; in fact, similar *transferability issues* have been reported previously in the literature. For example, see the incorrect 2D-3D transition in boron clusters for the borg-0-1 set^43^ and coordination dependence of repulsive potential for different polymorphs of ZnO.^16^ A similar trend in repulsive potentials for silicon polymorphs was also observed by Chou et al.^9^ They showed that the accuracy can be improved by increasing the cutoff of the repulsive potential from 3.5 to 6.3 Å (up to the fourth nearest neighbor for diamond). However, within the limits of the constraints used here, we see no significant improvement in extending the cutoff radii beyond 3.4 Å. It should be pointed out that Chou et al.^9^ also optimized the electronic parameters along with the repulsive potential.

We further note that there exists a repulsive potential for each individual polymorph that leads to an almost perfect agreement between the DFT and DFTB energies, see Figure 5. The variations in the shape of the repulsive potentials, across different polymorphs and with different constraints, can be appreciated by looking at Figure 5. Clearly, the repulsive curvature constraint leads to smoother repulsive potentials, but this comes at the expense of a slightly worse fit to the target energies in the training set. This is illustrated by the incorrect shape of the E-V curve for high-coordinated phases like SC and BCC. The aforementioned problem can be rectified by the use of a softer single minimum constraint (at most one minimum). The occurrence of attractive regions in the repulsive potential for condensed Si phases was also reported by Chou et al.^9^

The silicon example demonstrated here clearly indicates the problems in transferability of SCC-DFTB parametrizations. This issue will be discussed in more detail in the following.

#### 4.4.1 Transferability of the Repulsive Potential within a Polymorph

**Figure 6:**
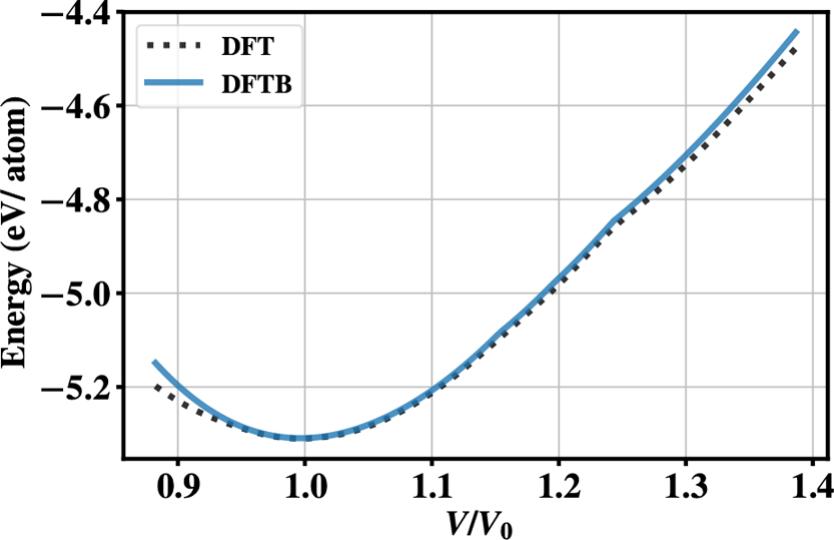
A comparison of energy-volume curve DFT (black) and SCC-DFTB (blue) for nonisotropic deformation of diamond.

The results from the above section show that it is possible to get a smooth repulsive potential that can describe the isotropic E-V curves for individual polymorphs. Here, we look at the transferability of the obtained repulsive potential for nonisotropic deformations. For this purpose, we consider E-V curves for the diamond (4C) structure along one axis keeping the other two constant. The results obtained are shown in Figure 6. The results indicate that the repulsive potential fitted on isotropic deformations is transferable for nonisotropic deformations.

### 4.5 Exploring Limits of Transferability Using Two-Body Repulsive Potentials

The results from Section 4.4 suggest that there does not exist a single repulsive energy expression (One-fits-all), consisting of one-body and two-body energy terms under the given constraints, that leads to a satisfactory fit across the various polymorphs of Si with different coordination numbers. At the same time, for each polymorph, we can readily obtain a repulsive potential which fits the data with minimal errors. Here, we will analyze the one-body and two-body contributions in detail, before discussing possible extensions of the method that could allow for a single energy expression to fit the whole data set.

As an example, let us consider the diamond (4C) and simple cubic (6C) structures of Si. We may express the two fitting approaches adopted so far in the following way. First, for the “One-fits-all” procedure, we may write
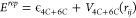
17where the subscripts indicate that there is a single one-body (ϵ) and single two-body (V) for both the 4C and 6C structures. Second, for the individual fits, we may write
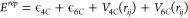
18where all energy terms are strictly zero when the coordination number does not match that of the subscript. From the previous sections, we know that the quality of the latter is superior to the former—but what about the other combinations of these ϵ’s and *V*’s? Using the same notation as before these would correspond to
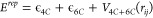
19a
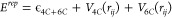
19b

**Figure 7:**
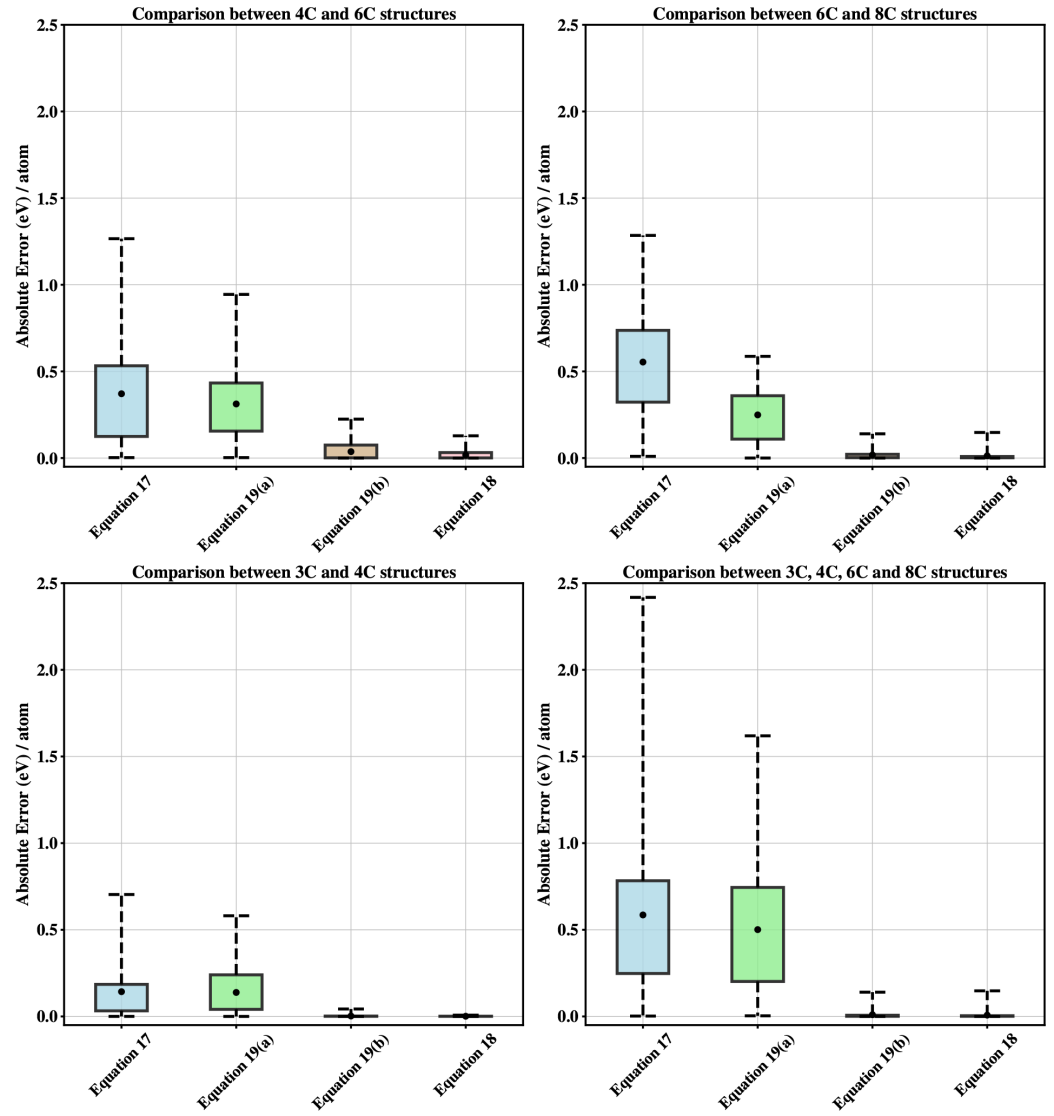
Absolute error per atom (*y* axis) for structures in the training set for different repulsive models presented in Section 4.5. The black dot indicates the mean absolute error. The upper and lower whiskers indicate the maximum and minimum errors for the training set.

Next, we consider a training set solely comprising of diamond (4C) and simple cubic (6C) E-V scans. We again use the siband Slater-Koster tables of Markov et al.^10^ for the electronic SCC-DFTB energies. Figure 7 shows a boxplot comparison using all four expressions above for repulsive fitting, with their combinations of ϵ’s and *V*’s for the 4C and 6C Si polymorphs. Additionally, we performed a similar analysis with other combinations of polymorphs corresponding to graphene + diamond and simple cubic + body centered cubic (see Figure 7).

Although the magnitude of the one-body term is large, there is little improvement in the fits when multiple ϵ’s are used compared to the corresponding fits with a single ϵ. Instead, the results indicate that we need to go beyond the simple two-body repulsive potential to reach transferability in SCC-DFTB. One such solution will be presented in the following section.

### 4.6 Beyond Two-Body Repulsive Potentials: Atomistic Neural Networks

It is clear that to describe a transferable repulsive potential with a single energy expression, we need to go beyond the one-body and two-body contributions. Ideally, we need a model that captures the local chemical environment with reasonable accuracy. Recently, machine learning models like Atomistic Neural Networks (ANN) have gained popularity for accurately describing the short-ranged interactions. However, a pure ANN approach fails to account for long-range interactions, even with large cutoff radii. In the case of SCC-DFTB, we have a good description of the long-range interactions but clearly lack transferability in the short-range description. The combination of the two is therefore appealing, and indeed such combined approaches have been presented in the literature using a Deep Tensor Neural Network together with SCC-DFTB.^23^

**Figure 8:**
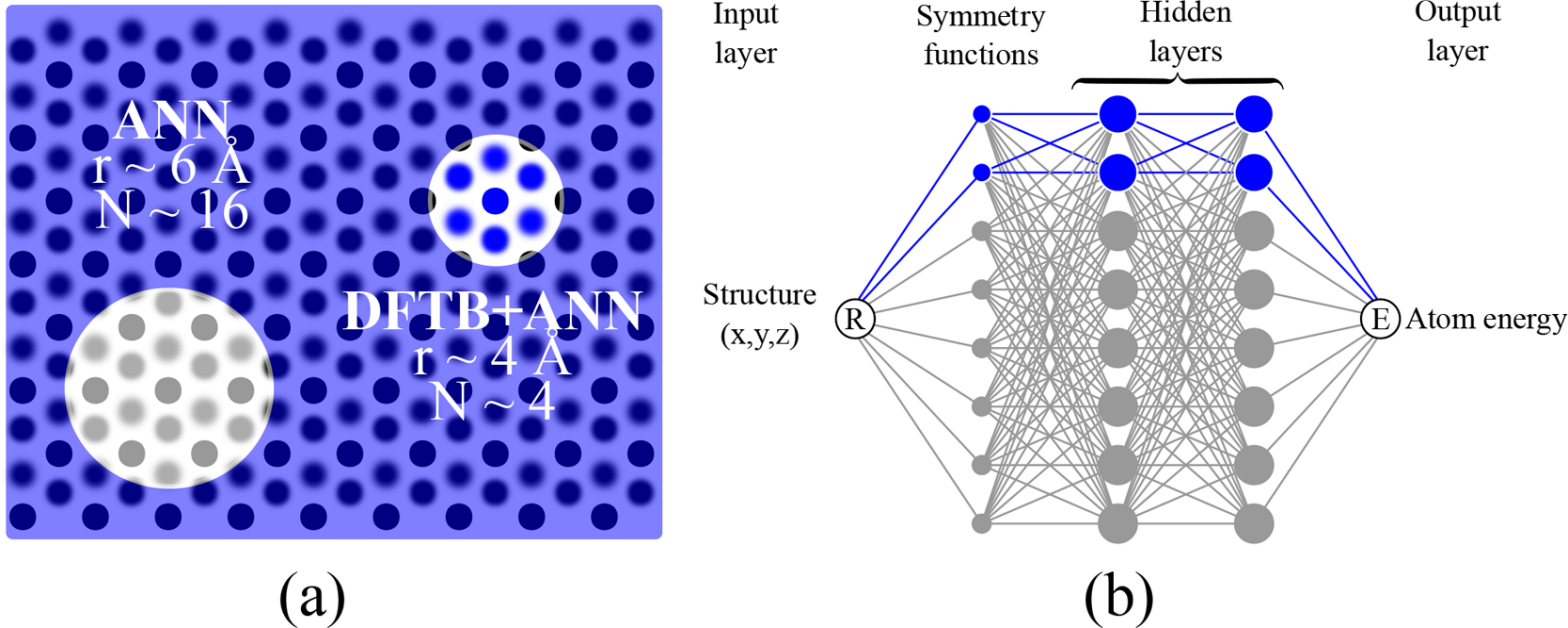
Schematic illustration of a conventional ANN (gray) and SCC-DFTB+ANN (blue) approach. The value of *r* indicates the radius of the cutoff sphere, and *N* is the expected number of neighboring atoms within the cutoff. The cutoff value can be kept small for the SCC-DFTB+ANN approach because of the inbuilt long-range interactions of the SCC-DFTB method.

**Figure 9:**
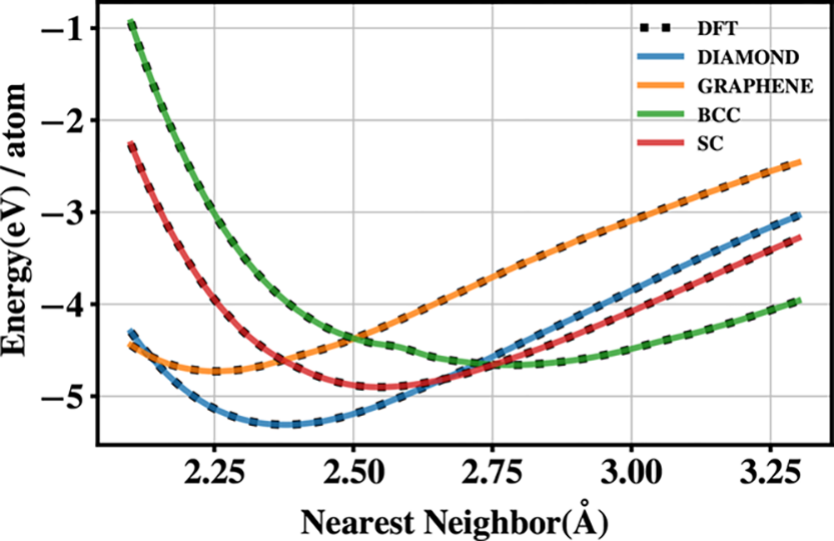
Comparison between DFT (black dotted lines) and SCC-DFTB+ANN energies for Si polymorphs using a neural network as the repulsive potential.

The Behler–Parinello Neural Network (BPNN)^44^ is a popular ANN architecture that has been proven to work well for molecular and solid systems. Here, we used a BPNN potential to approximate the repulsive potential. The general idea is to represent the local chemical environment of an atom using a set of radial and angular symmetry functions. Our network architecture comprises of four radial and four angular symmetry functions, two hidden layers with two nodes per layer, with a cutoff radius of merely 4 Å (typical cutoff radii are 6–10 Å^45^), and a hyperbolic tangent activation function. This is a much smaller and more nearsighted neural network representation as compared to pure ANN approaches. Figure 8 shows a schematic comparing a pure ANN approach and our DFTB+ANN approach. For the generation of the ANN potentials, we used the PROPhet package,^46^ which is an open source implementation of the BPNN method. The network was trained on the same data set (E-V curves of polymorphs) as used in section 4.4 and was optimized using a resilient backpropagation algorithm. The E-V scans for DFT and the corresponding SCC-DFTB+ANN methods are shown in Figure 9. Indeed, a nearsighted ANN repulsive potential can be used to get a “One-fits-all” repulsive potential. However, one should bear in mind that the increased transferability comes with a cost. The extrapolating power of using “chemically intuitive” functions is lost when using the ANN repulsive potential, which implies that the transferability of the repulsive potential to systems beyond the training set must be carefully investigated.

## 5 Conclusion

The goal in this work was to develop a fast and robust machinery to obtain SCC-DFTB repulsive potentials without having to resort to nonlinear fitting procedures. For this purpose, we used a scheme called CCS to generate the repulsive potentials. The CCS scheme was augmented with new constraints (repulsive and monotonous) and was successfully applied to create accurate repulsive potentials for Si polymorphs. The key features of the augmented CCS method include the following: *i)* Its ability to adopt various shapes without producing spurious oscillations, *ii)* The method is compatible with sparse data sets, and *iii)* The lack of hyperparameters reduces the global search/optimization space allowing us to fully concentrate on the electronic parameters. For individual polymorphs of Si, accurate parametrization of the repulsive potential was possible using CCS. However, due to the approximations in SCC-DFTB, global transferability is limited.

Regarding transferability, we show that a description beyond a two-body additive repulsive potential is required. In this respect, we further demonstrated that a nearsighted nonlinear ANN model can be a viable solution. The generalized repulsive potential approach by Kranz et al.,^21^ the multicenter tight binding approach of Goldman et al.,^25^ and the Deep Tensor Neural Network (DTNN) based many body repulsive potential of Stöhr et al.^23^ are steps along this path.
